# What’s God Got to Do With It? The Relationship Between Religion, Sadism, and Masochism

**DOI:** 10.5964/sotrap.13341

**Published:** 2024-09-30

**Authors:** Brooke Davis, Crystal Evanoff, Kelly M. Babchishin

**Affiliations:** 1 Department of Psychology, Carleton University, Ottawa, ON, Canada; 2 Department of Psychology, University of British Columbia, Kelowna, BC, Canada; Saint Mary's University, Halifax, NS, Canada

**Keywords:** sadism, masochism, religion, paraphilias

## Abstract

Although “BDSM” (i.e., bondage, discipline, dominance, submission, sadism, and masochism) has become increasingly present in popular media in recent years, much remains unknown about the etiology and correlates of BDSM. Research has demonstrated a relationship between religion and sexual behaviours/attitudes; therefore, religion could also be associated with sadism and masochism. To address gaps in existing knowledge, we conducted an online survey of 515 participants who answered a questionnaire on sexual life and behaviour, including questions on arousal in response to sadism and masochism scenarios, associated negative impacts, and religion. We found a higher prevalence of arousal in response to sadism scenarios amongst non-religious participants (64.6%; *n* = 228/353) than religious participants (54.7%; *n* = 88/161) with a small, but potentially meaningful effect size (Φ = -.095, *p* = .032). Increased impact of religious beliefs on sex life was associated with slightly lower sadism arousal, *r*(499) = -.080, *p* = .075. This association was strong enough to be considered a potentially meaningful factor but was not statistically significant. There was also a small negative correlation between masochism arousal and impact of religious beliefs on sex life and behaviour, *r*(500) = -.129, *p* = .004. Based on these findings, we conclude that there could be a limited but meaningful relationship between religion and sadism/masochism arousal. Further research should explore specific religious affiliations and beliefs as potentially associated with sadism and masochism arousal.

## Abstract (plain language summary)

**Background:**

Paraphilias are defined as sexual interest focused on an atypical activity or target. According to the World Health Organization, such interest can be considered “paraphilic” if it is persistent, intense, and paired with distress or behaviour that involves a significant risk of death or injury. Sadism (i.e., arousal in response to inflicting pain) and masochism (i.e., arousal in response to experiencing pain) are examples of such paraphilias that have become increasingly mainstream; however, there is still much that is unknown about these two paraphilias.

**Why was this study done?:**

Despite a growing understanding of the prevalence of sadism and masochism in the general population, gaps remain in the understanding of arousal related to paraphilias and paraphilic interests, including correlated demographic characteristics.

**What did the researchers do and find?:**

We asked an online sample consisting of undergraduate students and community participants about their sexual interests, their religious affiliation, and the impact of their religion on their sex life. Religious participants were less likely than non-religious participants to report arousal in response to sadism. Additionally, increased self-reported impact of religion on sex life was associated with decreased masochism and sadism arousal. The results indicated a high prevalence of arousal in response to sadism (62%) and masochism (76%) amongst sample participants.

**What do these findings mean?:**

The findings of this research suggest that there could be a weak negative relationship between religion and arousal in response to sadism and masochism. These findings also suggest that interest in sadism and masochism arousal is common in the general population, with more than half of the participants indicating at least some arousal in response to sadism and masochism. The methods applied in this study limit its generalizability. The majority of participants were non-religious, which is not reflective of the general population from which the data were drawn. Additionally, the sample used in this study was one of convenience and could, therefore, be meaningfully different from the general population. The current study was cross-sectional, so cause and effect cannot be determined. More research is needed to better understand the relationship between religion and these two paraphilias.

## Highlights

- 515 student and online participants responded to an online survey related to sexual orientation, arousal, fantasies, masturbation, and demographic information.
- Sadism arousal was slightly more prevalent for non-religious participants than religious participants.
- More self-reported impact of religion on sex life was related to lower masochism and sadism arousal.

While bondage, discipline, sadism, and masochism, often referred to as BDSM, have become increasingly mainstream in recent years, widespread acceptance and understanding are lacking (e.g., Freeburg & McNaughton, 2017). Accordingly, there is still much that is unknown about the two sexual paraphilias related to BDSM: sadism and masochism. There is a growing body of evidence to suggest that sexual interest in sadism and masochism is both prevalent in the general population (e.g., Bártová et al., 2021) and not meaningfully related to mental health outcomes (e.g., Brown et al., 2023). Research with community samples has reported varying rates of prevalence of sexual interest in sadism ranging from about 7% to 72% (Median [*Mdn*] = 12.8%; Ahlers et al., 2011; Bártová et al., 2021; Dawson et al., 2016; Holvoet et al., 2017; Joyal & Carpentier, 2017; Seto et al., 2021). Similarly, reported prevalence of sexual interest in masochism also varies, ranging from about 15% to 69% (*Mdn* = 20.4%; Ahlers et al., 2011; Bártová et al., 2021; Dawson et al., 2016; Holvoet et al., 2017; Joyal & Carpentier, 2017; Seto et al., 2021).

Paraphilia is defined as an atypical sexual interest greater than or equal to nonparaphilic sexual interests (i.e., “sexual interests in genital stimulation or preparatory fondling with phenotypically normal, physically mature, consenting human partners”, *Diagnostic and Statistical Manual of Mental Disorders: Fifth Edition, Text Revision* [American Psychiatric Association, 2022; DSM-5-TR, p. 779]). The prevalence of sadism and masochism paraphilias in community samples has been found to be lower than the prevalence of interest in sadism and masochism activities. For example, one study found that between 0.3% and 1.2% of a sample of adults that was representative of the Québec population (a large Canadian province) had a sadism paraphilia, whereas between 1.4% and 4.9% had a masochism paraphilia (Joyal & Carpentier, 2017). A study conducted in Australia found that 1.8% of sexually active people had engaged in BDSM behaviour in the past year (Richters et al., 2008); however, behaviours do not necessarily indicate paraphilic interest or disorder. Paraphilic disorders are identified and defined in the World Health Organization’s (2019) *International Statistical Classification of Diseases and Related Health Problems,* (11^th^ edition; ICD-11) as persistent and intense atypical sexual arousal. This atypical sexual arousal is considered a disorder if the person is distressed by this arousal or the paraphilic behaviour involves a significant risk of death or injury (World Health Organization, 2019).

There is a limited understanding of the correlates of paraphilic interests. Research suggests that increased interest in certain paraphilias could be related to multiple factors, such as sex drive (Dawson et al., 2016), psychopathic traits (Brown et al., 2023), and attitudes supporting paraphilias (Brown et al., 2023). Research also suggests that paraphilia-related fantasies can start in childhood (Cantor, 2012) and that there is a relationship between childhood experiences, such as abuse, especially sexual abuse, and paraphilic interests in adulthood (e.g., Abrams et al., 2022; Pedneault et al., 2020).

## Religion and Sexuality

Research has previously demonstrated relationships between religion and sexual attitudes and behaviours. Therefore, religion could be a source of further information about psychological factors underlying arousal in response to paraphilias. For example, there is evidence that increased religiosity, or the extent of one’s involvement in religion, is associated with more sexually conservative attitudes (Ahrold et al., 2011). Intrinsic religiosity (*r* = -.26, *p* < .01; Marcinechová & Zahorcova, 2020) and spirituality (*r* = -.43, *p* < .001; Murray et al., 2007) have both been found to be negatively correlated with sexual permissiveness. Furthermore, rules related to sexuality appear to be of particular importance in religious morality, as religious people rate violations of sexual morality as worse than violations of cooperation morality, such as cheating on taxes (Hone et al., 2021).

Religious fundamentalism, intrinsic religiosity, spirituality, and religious beliefs have been shown to be positively related to “sex guilt” (Emmers-Sommer et al., 2018; Woo et al., 2012), which is defined as guilt experienced as a result of “violating or anticipating violating standards of proper sexual conduct” (Mosher & Cross, 1971, p. 27). This could be related to a more general relationship between religion and shame, as research has found that negative religious coping strategies (e.g., feeling punished by God) are associated with increased shame proneness (Ladis et al., 2023). However, research to date does not universally support the theory that religion is positively associated with shame around sex. For example, one study exploring the relationship between shame, guilt, and hypersexual behaviours noted that there was no relationship observed between religious affiliation and guilt as a result of hypersexual behaviours (Gilliland et al., 2011).

### Religion and Paraphilias

Historically, religion was a key consideration in defining what was “sexually deviant” (De Block & Adriaens, 2013). While much has changed regarding acceptable sexual practices, sexual morality continues to be a part of some religious belief systems. For example, some Christian religions emphasize the importance of moderate sexual relations and prohibit sexual practices focused on pleasure rather than procreation (Johnson & Jordan, 2006). Therefore, it is possible that religious affiliation could influence its members’ sexual interests and behaviours, including with regard to sexual paraphilias, such as sadism and masochism.

Common religious themes, such as obedience and submission to dominant authority figures, in contrast, are reminiscent of elements of sadism and masochism (e.g., Hamman, 2000). As such, religious people may be more interested in or open to sadism and masochism than non-religious people. In recognition of some of the evident similarities between masochism and certain religious practices (e.g., self-flagellation), the DSM-5-TR (American Psychiatric Association, 2022) highlights the importance of not conflating the two. There is also some indication that people can have spiritual experiences during BDSM acts (e.g., Baker, 2018), suggesting another possible positive association between religion, sadism, and masochism.

Alternatively, there may be an inverse relationship between religion, sadism, and masochism (i.e., religious people having less interest). A recent study found that religiosity was positively associated with negative attitudes about BDSM (Grigoropoulos, 2022). Ruppelius (2019) found evidence that paraphilic interests and behaviours are associated with distress for people who are higher in religiosity; however, the generalizability of this finding is limited as participants were exclusively college students in one geographic location (Hawaii). Therefore, a relationship between religion, sadism, and masochism could also exist due to non-supportive attitudes about these behaviours or distress experienced by religious people when their sexual fantasies conflict with their religiously rooted attitudes and beliefs about sex.

## Current Study

This study aims to contribute to the body of research on sadism and masochism arousal and paraphilic interests in the general population. In particular, we examined the characteristics of those with a sexual interest in sadism and/or masochism and explored the nature of the relationship between religion, sadism, and masochism in an online survey of 515 participants.

## Method

### Participants

A total of 309 students and 584 online participants started the online survey. Participants were excluded from this study if they did not complete the survey (*n* = 191), indicated they had been less than fully honest (*n* = 173), or did not respond to the questions on sadism or masochism (*n* = 14). A total of 515 participants met these criteria (57.7% of the total); however, one participant did not respond to the necessary questions on sadism arousal. Participants in this study overlap with an earlier study that examined the link between paraphilic interests and sexual and life satisfaction (see Mundy & Cioe, 2019).

Participants in this study were predominantly female (67%) and straight (63%) and reported having a variety of ethnicities, with European being the most commonly reported ethnicity (42%). The average reported age of participants was 24 years old. Detailed characteristics of participants for the sadism and masochism samples are included in Table 1.

**Table 1:** Sample Characteristics (n = 515)

Sample	
Student	48.9%
Online	51.1%
Sex assigned at birth	
Male	33.0%
Female	67.0%
Religion	
None	68.7%
Christian, Orthodox	4.1%
Christian, Roman Catholic	9.5%
Christian, Evangelist	4.7%
Jewish	0.8%
Islamic	1.2%
Hindu	1.0%
Buddhist	1.2%
Other	8.9%
Ethnicity	
Aboriginal	1.2%
African	1.4%
Asian	10.3%
Caribbean	0.8%
European	42.1%
Latin, Central, and South American	3.1%
North American	28.5%
Oceania	4.9%
Other^a^	7.2%
Not applicable	0.6%
Age^b^	
< = 19	28.2%
20-29	57.2%
30-39	9.6%
40-49	3.3%
50-59	1.5%
60-69	0.2%
Sexual orientation^c^	
Straight	84.4%
Not straight	15.6%

^a^The other category was not defined. ^b^36 participants did not provide their age. ^c^One participant did not respond to the question on sexual orientation.

### Measures

The Sexual Life and Behaviour Questionnaire (SLBQ; Ahlers et al., 2011) includes 87 sections of questions related to sexual orientation, arousal, fantasies, and masturbation, along with questions on demographic information, including religion, age, and sexual orientation. The questions of interest for this analysis asked participants to rate their level of sexual arousal in response to sadism/masochism (“How sexually arousing do you find it when your sexual partner is dominant [e.g., handcuffs you, causes you pain]?” / “How sexually arousing do you find it when you are dominant (e.g., you handcuff your partner, you cause pain)?”) and report the duration of this fantasy. Participants were also asked about their normative sexual interests (“How sexually arousing do you find adult women and the adult female body [fully developed figure, developed pubic hair, breasts]?” / “How sexually arousing do you find adult men and the adult male body [fully developed masculine figure, developed pubic hair, genitalia]?”). Response options for sexual arousal questions were “Not at all,” “Slightly,” “Moderately,” “Very,” or “Extremely.” If participants indicated at least some interest in the paraphilia, they were asked two questions on related negative impact (“How often are you negatively affected by this fantasy?” and “How often does this fantasy negatively impact your relationship, social, and work life?”). Response options were “Never,” “Rarely,” “Sometimes,” “Often,” and “Almost always.”

#### Religion

Participants were also asked if they belonged to a religion and were provided with the following response options: “No,” “Christian, Orthodox,” “Christian, Roman Catholic”, “Christian, Evangelist,” “Jewish”, “Islamic”, “Hindu,” “Buddhist”, and “Other, please specify.” Participants also indicated the extent to which their religion impacts their sex life (“How much do your religious beliefs impact your sexual life and sexual behaviour?”). Response options were “Not at all,” “Slightly,” “Moderately,” “Very,” or “Extremely.”

### Procedure

Participants for this study were recruited in an earlier study (Mundy & Cioe, 2019) from an undergraduate participant subject pool and an online survey. Community sample participants were recruited via a recruitment poster that described the purpose of the study (to understand sexual interests) and provided the link to the questionnaire on online forums (r/sex, r/SampleSize and r/TwoXChromosomes). After completing the questionnaire, participants were asked to rate their honesty, provided a debriefing form, and provided the link either to enter a draw for a gift card (1 in 39 chances of winning $50 gift card; community sample) or to submit their name for course credit (university pool). To address risks related to participants possibly disclosing criminal activities, participants were informed about information storage and applicable laws and advised that they could withdraw at any time without penalty. Efforts were taken to protect participants’ identities, including not requesting any identifying information. Ethics approval for the original study was obtained (H15-00752). Ethics approval was not required for the current study as it involved secondary data analysis of anonymized data.

### Data Analyses

#### Indices

Three thresholds of interest in sadism and masochism were considered in this analysis: any interest, paraphilic interest, and paraphilic disorder, as defined by the DSM-5-TR (American Psychiatric Association, 2022). We considered an interest paraphilic if the level of arousal was intense, persistent, and greater than or equal to arousal in response to normative sex. We considered paraphilic interest to be a disorder if it was coupled with distress or impairment of important functioning. We verified that none of the participants considered to have paraphilic interest/disorder had reported no arousal in response to normative sex and no arousal in response to sadism or masochism.

Participants were considered “straight” if they indicated that their assigned gender at birth was male and that they were interested in exclusively or mostly women or if they indicated that their assigned gender at birth was female and that they were interested in exclusively or mostly men. Male participants were considered to be “not straight” if they indicated that they were interested in both men and women, mainly or exclusively men or other. Additionally, female participants were considered to be “not straight” if they indicated that they were interested in both men and women, mainly or exclusively women or other.

Participants were considered to have at least some interest in sadism (*n* = 316) or masochism (*n* = 393) if they selected “Slightly,” “Moderately,” “Very,” or “Extremely” in response to the relevant sadism or masochism arousal questions. Participants were considered to have a paraphilic interest in sadism (*n* = 54) or masochism (*n* = 122) if their level of reported arousal was intense (i.e., “Very” or “Extremely”), greater than or equal to their highest reported arousal in response to adults, and started at puberty/first sexual experience or more than six months ago. Participants were considered to have sadism (*n* = 22) or masochism (*n* = 57) disorder if they met the threshold for paraphilic interest and also reported at least some negative impact (i.e., they selected “Rarely,” “Sometimes,” “Often,” or “Almost always” for either of the two questions on negative impact).

Due to sample size limitations, religious affiliation was treated as a dichotomous variable. Participants who selected any religion options (*n* = 161) were included in the religion group, and the rest were included in the no religion group (*n* = 354).

#### Analyses

Analyses were conducted using SPSS version 28.0.1.1 (version 14) and RStudio version 2023.06.2+561. *T*-tests were conducted to determine if average arousal in response to sadism and masochism for religious and non-religious participants was statistically significantly different. Cohen’s *d* was calculated to determine if the effect size was small (.2), medium (.5) or large (.8; Cohen, 1992). Chi-squared statistics were calculated to compare prevalence across religious affiliation groups on any interest, paraphilic interests and negative impact for both sadism and masochism. Phi (Φ) was calculated to determine if effect sizes were small (.1), medium (.3) or large (.5; Cohen, 1992). The relationship between the reported impact of religion on sex life and arousal in response to sadism and masochism was assessed using Pearson correlation coefficients. A Pearson correlation coefficient of .1 was considered a small effect, .3 was considered a medium effect and .5 was considered a large effect (Cohen, 1992). As these are arbitrary cut-offs, we considered Cohen’s *d* of +/- .15 a meaningful effect size for identifying individual risk factors (Mann et al., 2010), which corresponds to an *r* of +/-.075 and phi of +/-.075.

As an exploratory analysis, we applied a hierarchical linear regression analysis to sadism and masochism separately to explore the extent to which variability in arousal was explained by religion when controlling for key demographic information. The first model assessed the relationship between religion (affiliation and impact of religion on sex life) and arousal. This model was compared to a second model that added two potential covariates, age and sexual orientation, in order to control for any relationship those variables have with religion and arousal in response to sadism and masochism.

## Results

### Prevalence

Most participants reported some arousal to scenarios of sadism (61.5%; 316/514) and masochism (76.3%; 393/515). About one in 10 participants had a paraphilic interest in sadism (i.e., interest in sadism is equal to or higher than normative sexual interest; 10.5%; 54/513) and approximately one in four participants had a paraphilic interest in masochism (23.8%, 122/513). Finally, about one in 20 participants in this sample had sadism paraphilic disorder (i.e., paraphilic interest also reporting distress; 4.3%, 22/513), and about one in 10 participants had masochism paraphilic disorder (11.1%, 57/513).

While there was variation in the prevalence of sadism and masochism paraphilic interests across several variables, many differences did not reach the threshold for statistical significance (see Davis et al., 2024S, Table S1). There were, however, small statistically significant differences across sample type (student vs. online), sex assigned at birth, and sexual orientation.

### Religious Affiliation

**Figure 1:**
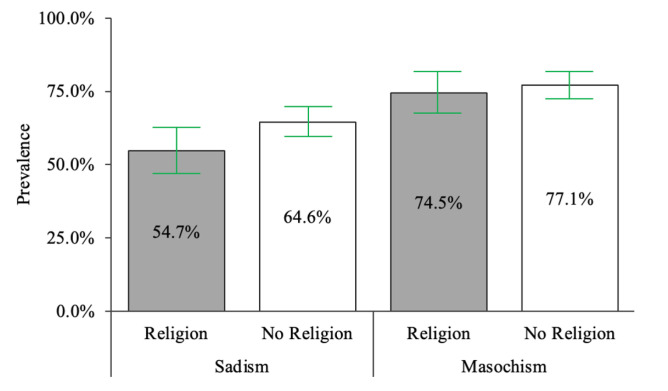
Prevalence of Any Arousal in Response to Sadism and Masochism *Note.* 95% confidence intervals were calculated for each sample proportion.

The relationships between sadism and masochism and religious affiliation generally had small effect sizes. Non-religious participants were slightly more likely to report any arousal in response to sadism (64.6%, 228/353) than religious participants (54.7%, 88/161), χ^2^(1, *n* = 514) = 4.61, *p* = .032, Φ = -.095; see Figure 1. This relationship reached the conventional statistical significance threshold and the Mann et al. (2010) threshold for a meaningful risk factor. Non-religious participants (77.1%, 272/354) had similar arousal to masochism than religious participants (74.5%, 120/161); the difference was not statistically significant, and the effect size was small, χ^2^(1, *n* = 515) = .409, *p* = .523, Φ = -.028, and below the Mann et al. (2010) threshold of a meaningful factor.

Average reported arousal in response to sadism was higher for non-religious participants (x̄ = 2.34, σ = 1.26) than religious participants (x̄ = 2.14, σ = 1.26), *t*(512) = 1.723, *p* = .086, *d* = .16. While not statistically significant, this effect size was larger than the Mann et al. (2010) threshold for a meaningful factor. The average reported masochism arousal was similar for non-religious participants (x̄ = 3.02, σ = 1.43) and religious participants (x̄ = 2.84 σ = 1.43); this effect was smaller than the Mann et al. (2010) threshold for a meaningful factor, *t*(513) = 1.29, *p* = .197, *d* = .12, and not statistically significant.

The results of the hierarchical linear regression analysis suggest that age and sexual orientation did not influence the relationship between religion and sadism and masochism arousal (see Davis et al., 2024S, Tables S3 and S4).

### Paraphilic Interests

Non-religious participants had a similar level of paraphilic interest in sadism (11.1%, 39/352) to religious participants (9.3%, 15/161), χ^2^(1, *n* = 513) = .36, *p* = .546, Φ = -.027. Similarly, non-religious participants had similar paraphilic interest in masochism (24.4%, 86/352) to religious participants (22.4%, 36/161), χ^2^(1, *n* = 513) = .26, *p* = .609, Φ = -.023. None of these analyses reached conventional thresholds for statistical significance or met the threshold for a meaningful factor by Mann et al. (2010).

### Reporting Experiencing Negative Impact

The prevalence of any negative impact experienced as a result of sadism and masochism arousal was compared across religious and non-religious participants. A negative impact of sadism arousal was slightly more prevalent among non-religious participants (26.0%; 59/227) than religious participants (18.0%; 16/89). This difference did not reach statistical significance convention but did reach meaningful effect as per Mann et al. (2010), χ^2^(1, *n* = 316) = 2.268, *p* = .132, Φ = -.085. Non-religious participants did not differ on their reported negative impact as a result of masochism-related arousal (30.4%, 83/273) compared to religious participants (28.3%, 34/120), χ^2^(1, *n* = 393) = .171, *p* = .679, Φ = -.021.

### Impact of Religion on One’s Sex Life

In addition to the role of religious affiliation, religion's importance in participants’ sex lives was also considered to see if it was related to sadism and masochism. Of participants who responded to the related question (*n* = 501), sadism arousal was weakly and negatively correlated with the impact of religious beliefs on sex life and behaviour, *r*(499) = -.080, *p* = .075. This correlation is slightly higher than the Mann et al. (2010) threshold for a meaningful factor but was not statistically significant. For participants who responded to the relevant questions for masochism (*n* = 502), there was a statistically significant negative correlation between the level of arousal and the impact of religious beliefs on sex life and behaviour, *r*(500) = -.129, *p* = .004. This correlation was both statistically significant and large enough to be considered meaningful.

## Discussion

We found a limited relationship between religion and arousal in response to sadism and masochism. The effect sizes for the observed associations were small compared to Cohen’s thresholds (1992). However, given the complexity of sexuality and the likely large number of contributing factors, we would not expect individual factors to have large or even moderate effects. Informed by research on risk factors (Mann et al., 2010), we would argue that individual variables should be considered meaningful contributors to understanding sexuality at lower thresholds than the general levels specified by Cohen (1992). Several small effect sizes observed in this study surpass the Mann et al. (2010) threshold for meaningful risk factors, suggesting that religion may provide some helpful information on arousal in response to sadism and masochism. Specifically, we found a higher prevalence of self-reported arousal in response to sadism scenarios amongst non-religious participants compared to religious participants and a non-significant but potentially meaningful negative correlation between impact of religion on sex life and sadism arousal. Additionally, while we found no meaningful relationship between religious affiliation and masochism arousal, we observed a negative association between the impact of religion on sex life and masochism arousal. That is, as participants’ self-reported impact of their religious beliefs on their sex life increased, their reported level of masochism arousal decreased. While the findings suggest a negative association between religion and both sadism and masochism, it is not clear why the findings were different for sadism and masochism (i.e., sadism arousal is potentially related to both religious affiliation and impact, while masochism is only related to impact).

The observed direction of the relationship between religious affiliation and the self-reported negative impact of both sadism and masochism arousal (i.e., religious affiliation was associated with a lower prevalence of negative impact), while not significant, was negative. This was not expected given previous research (e.g., Ruppelius, 2019) and also did not align with the potential explanation that religious participants report less arousal from sadism and masochism due to discomfort. However, participants only responded to negative impact questions if they indicated some arousal; consequently, participants who denied any arousal, perhaps due to associated distress, would not be included. Therefore, it is possible that the relationship between religion and negative impact of sadism and masochism is not fully captured in this analysis.

While not the focus of this study, it is worth noting that straight participants were statistically significantly less likely to have a paraphilic interest in masochism than non-straight participants. Further exploration is needed as this result could be due to sexual openness expressed both as openness to a variety of sexual partners and a variety of sexual practices. However, this does appear to be consistent with previous research that found higher rates of recent BDSM behaviours amongst bisexual, gay, and lesbian people than straight people (Richters et al., 2008).

Sexual arousal and behaviours are likely complicated and influenced by a number of variables. Although we have not controlled for all possible confounding variables, we did find an association between arousal in response to sadism and masochism and two potential confounding variables: sexual orientation and age. However, adding these variables to the model did not meaningfully change the nature of the relationship between religion and arousal in response to sadism and masochism.

Religiosity has been found to influence sexuality, for example, by being associated with more conservative attitudes (Ahrold et al., 2011) and less sexual permissiveness (Marcinechová & Zahorcova, 2020; Murray et al., 2007). Given that religion seems to influence sexuality variables, it follows that it could also influence atypical sexualities, such as sadism and masochism. In the current study, we found a weak negative relationship between religion and arousal in response to sadism and masochism. Additional research suggests that involvement in church and belief in religion could facilitate desistance in men with sexual offences (Harris et al., 2017) and crime in general (e.g., Pirutinsky, 2014). Religious affiliation’s potential association with paraphilias (as a possible protective factor) warrants further exploration.

### Limitations and Future Research Directions

This is a modest study using cross-sectional design and a convenience sample of online participants and students. A number of factors limit the broad applicability of the findings of this study. First, participants’ interest in sadism and masochism was determined exclusively with information gathered via self-report. A recent meta-analysis on the role of social desirability in self-reporting of sexual behaviours found correlations between social desirability and underreporting of a number of sexual behaviours (e.g., same-sex relations, anal sex, and extramarital affairs; King, 2022). While this is a common challenge in sex research with a community sample, it is potentially especially challenging in the context of the research questions explored in this study. Previous research suggests that religiosity is associated with impression management (Gillings & Joseph, 1996), and intrinsic religiosity is correlated with social desirability (Trimble, 1997). There is also evidence that religiosity is negatively correlated with acknowledging pornography use (Grubbs et al., 2015). However, studies conducted to explore the role of social desirability in self-reporting of pornography consumption and virginity found no difference in socially desirable responding between religious and non-religious participants (Rasmussen et al., 2018; Regnerus & Uecker, 2007). Therefore, it is not clear how religion impacts the self-disclosure of sexual behaviours/interests generally or how it would impact self-disclosure related to paraphilic arousal or behaviours.

As such, religious people could experience less arousal in response to masochism and sadism. Alternatively, it could be that religious people are less willing to report arousal in response to masochism and sadism, perhaps because this arousal is viewed as undesirable. To address the challenge of socially desirable responding, future research should consider including indirect measures of sexual interest (Pedneault et al., 2021). For example, applying the bogus pipeline method, which has been shown to increase the accuracy of self-reporting of sexual arousal (e.g., Suschinsky et al., 2020), measuring viewing time (e.g., Schmidt, Babchishin, & Lehmann, 2017), or using methods such as video clips to depict activities while assessing viewing time (Lalumière et al., 2018), could help to address this challenge.

The prevalence of religious affiliation in this sample also limits the applicability of the findings of this study. As only one-third of participants reported having a religion, there was not a sufficient number of participants in each religion category to explore differences between religious groups. If there is a relationship between religion, sadism or masochism, it is likely to vary depending on the nature and importance of the specific religious teachings about sexuality. For example, Hinduism, which has been identified as a religion that tolerates “deviance” (Bhugra et al., 2010), would likely have a different impact on paraphilic interests than a more socially conservative religion. Therefore, the approach taken in this study could have missed meaningful relationships between specific religions, sadism, and masochism. Recruitment of a larger sample with a sufficient representation of religions would help to address these issues.

Furthermore, questions in this study used to measure participants’ religion focus on participants’ current religious affiliation and reported impact of religion (e.g., “Do you belong to a religion?” and “How much do your religious beliefs impact your sexual life and sexual behaviours?”). However, previous research suggests that childhood experiences can be related to adult interest in paraphilias (e.g., Abrams et al., 2022). Therefore, childhood religious influences, which would not be captured in this study, may be more relevant to arousal in response to sadism and masochism than current religious involvement. Future research can address this limitation by including measures of childhood religion.

The rate of religious affiliation in this sample is very low compared to the prevalence in Canada (the sample’s origin), which was 68.3% in 2019 (Cornelissen, 2021). This suggests that the sample for this study is not representative of the Canadian population in terms of religion. The average age of participants was also young (24 years old), so findings may not generalize to older populations. As the importance of religious belief decreases as birth year increases amongst Canadians (Cornelissen, 2021), a younger sample, such as the sample in this study, could be less influenced by religion, including with regard to sexual arousal and behaviour. A larger, more representative sample would address this challenge.

We did not have a representative sample, so the prevalence rates in our sample are not likely representative. Indeed, the prevalence of any arousal in response to sadism (61.5%) was at the high end of the expected range based on previous research (e.g., Ahlers et al., 2011; Bártová et al., 2021; Dawson et al., 2016; Holvoet et al., 2017; Joyal & Carpentier, 2017; Seto et al., 2021), and the prevalence of masochism arousal (76.3%) was notably higher than the observed prevalence in previous research (e.g., Ahlers et al., 2011; Bártová et al., 2021; Dawson et al., 2016; Holvoet et al., 2017; Joyal & Carpentier, 2017; Seto et al., 2021). It is possible that this sample is unique in some way and, therefore, not in line with samples from previous research or representative of the general population. For example, 51.0% of participants were recruited from predominantly sex-oriented websites. Alternatively, the observed higher prevalence could be due to the wording of the questions used to gather information on arousal in response to sadism and masochism, which were broad and used relatively mild examples of these paraphilias (e.g., one example of sadism was “you handcuff your partner”). Additionally, the generalizability of our findings could be limited by bias introduced as a result of participants self-selecting into the study (e.g., Andrade, 2020).

### Concluding Remarks

Religious participants reported slightly less arousal in response to sadism, and the reported impact of religion on sex life was negatively associated with sadism arousal. For masochism, only the impact of religion on sex life was negatively associated with arousal. Based on these findings, we conclude that there could be a limited but potentially meaningful relationship between religion and sadism/masochism arousal. Future research using more representative samples and more objective measures would be needed to understand what role, if any, religion has in these seemingly mainstream paraphilic interests.

## Supplementary Materials

The Supplementary Materials contain the following items (for access, see Davis et al., 2024S):

- Table S1: summarizing the characteristics of participants with paraphilic interests
- Table S2: summarizing the outcome of a hierarchical linear regression on the relationship between religion and sadism arousal when controlling for key demographic information
- Table S3: summarizing the outcome of a hierarchical linear regression on the relationship between religion and masochism arousal when controlling for key demographic information

DavisB.
EvanoffC.
BabchishinK. M.
 (2024S). Supplementary materials to "What’s God got to do with it? The relationship between religion, sadism, and masochism"
[Additional information]. PsychOpen. 10.23668/psycharchives.15244
PMC1193912940143992

## Data Availability

The research data that support the findings of this study is available upon request.
